# Physiologically Constrained Deep Learning in Physiologically Based Pharmacokinetic Modeling: Virtual Patient Generation, Machine-Learned Scaling Laws, and Pediatric Precision Dosing

**DOI:** 10.3390/pharmaceutics18101256

**Published:** 2026-10-03

**Authors:** Lei Zhang, Yichao Xu

**Affiliations:** 1 Department of Pharmacy, The Second Affiliated Hospital, Zhejiang University School of Medicine, Hangzhou 310009, China; 2 Center of Clinical Pharmacology, The Second Affiliated Hospital, Zhejiang University School of Medicine, 88 Jiefang Road, Hangzhou 310009, China

**Keywords:** physiologically based pharmacokinetic modeling, virtual patients, generative deep learning, pediatric precision dosing, antiepileptic drugs

## Abstract

Physiologically based pharmacokinetic (PBPK) modeling is now central to pediatric dose selection, because clinical sampling in children is ethically constrained and simple weight-based scaling is unreliable while organ function and enzyme activity are still maturing. Three bottlenecks limit classical PBPK in this setting, namely building physiologically coherent virtual patient populations, extrapolating drug disposition across species and age, and estimating many mechanism-linked parameters from sparse data. Over the past five years, deep learning has begun to address each. Generative models constrained by physiological knowledge create virtual patients that preserve realistic correlations and whole-body coherence. Graph networks and neural ordinary differential equations learn continuous scaling and maturation relationships instead of relying on fixed exponents. Physics-informed networks and surrogate emulators estimate mechanistic parameters and quantify uncertainty in data-poor pediatric settings. This review surveys these developments, organizes them by the PBPK workflow they serve, and links them to precision dosing of three antiepileptic drugs, lacosamide, lamotrigine, and oxcarbazepine. Unlike earlier surveys focused on concentration prediction and therapeutic drug monitoring, we keep the mechanistic model in the foreground, treating the virtual patient as the unit of simulation. We also discuss open problems, including validation standards for generated populations, the regulatory qualification of learning-enhanced PBPK, uncertainty reporting, and the prospect of foundation models of pharmacokinetics. Throughout, we distinguish methodological maturity from demonstrated clinical benefit.

## 1. Introduction

Children are not small adults. The aphorism is repeated so often in regulatory documents and review articles that it risks losing its force, yet it names a real and long-standing difficulty in pediatric pharmacology. Growth and maturation reshape body composition, organ weights, blood flows, the expression of enzymes and transporters, and renal function. These changes are nonlinear, rapid during specific developmental windows, and only partially characterized [1,2,3]. Randomized trials in children are limited by ethics, recruitment, and sparse sampling. The evidence base for pediatric dosing has therefore accumulated mostly by taking adult data and scaling it down empirically. Those failures are well documented, from underexposure in infants to concentration-dependent toxicity in young children [1,2], and the evidence stays thin.

Physiologically based pharmacokinetic (PBPK) modeling came into being largely to replace that empiricism with mechanism. A PBPK model represents the body as organs and tissues connected by blood flow, each with a defined physiology. Drug-specific properties, such as partition coefficients, permeability, intrinsic clearance, and protein binding, are integrated through mass-balance ordinary differential equations [3,4]. The physiological layer, with its organ volumes, blood flows, and tissue composition, is shared between species and across ages. This sharing gives PBPK a distinctive advantage for extrapolation, because drug disposition can be predicted from animals to humans or from adults to children by swapping physiological inputs rather than re-estimating the drug model [3,5]. The advantage is one of mechanism rather than of exclusivity. Population pharmacokinetic (PopPK) models that embed allometric scaling and maturation functions are also used for pediatric extrapolation, and they typically require less prior information, but the dependence of clearance on age must be specified empirically and cannot be interrogated for a mechanism the data do not contain [6,7,8]. What distinguishes PBPK is that the physiological layer is represented explicitly, so that moving to a new age or species changes the model inputs rather than the estimated parameters. Regulators now accept PBPK as a quantitative tool for pediatric dose selection, and pediatric PBPK analyses have become routine components of pediatric investigation plans and pediatric study plans [9,10].

Maturity has not removed three bottlenecks that constrain PBPK in pediatric development. Virtual patient populations are the simulated cohorts in which pediatric doses are evaluated. They are usually assembled by sampling physiological parameters from marginal distributions, imposing correlation structure only partially, and enforcing biological coherence through ad hoc rejection sampling, for instance total organ mass not exceeding body weight [11,12]. Cross-species and cross-age extrapolation still depends on empirical allometric exponents, about 0.75 for clearance, and on ontogeny functions fitted piecewise to sparse proteomic data. Both are widely acknowledged to be fragile [13,14,15]. PBPK models are also parameter-dense. Estimating or updating mechanism-linked parameters, such as enzyme abundances, partition coefficients, and transporter activities, from limited clinical data remains a difficult inverse problem. It is tackled classically through manual calibration or Monte Carlo searches [16,17]. None of the three bottlenecks is new, and all remain open.

Over the past five years deep learning has begun to touch each of these bottlenecks, coalescing into a research program we call physiologically constrained deep learning for PBPK. We deliberately use the two near-synonyms in a specific way. Physiology-informed describes models whose loss function carries physiological knowledge (physics-informed losses, mechanistic residuals). Physiologically constrained describes models whose outputs are required, by construction or by hard constraints, to be physiologically admissible. One disciplines the training objective; the other disciplines the generated sample. Generative models learn the multivariate, developmentally structured distribution of human physiology and can sample virtual patients that satisfy hard physiological constraints [11,18,19]. Graph networks coupled with neural ordinary differential equations learn continuous allometric and ontogeny relationships straight from data, going beyond fixed exponents [13,18,20]. Physics-informed neural networks and surrogate emulators estimate parameters, propagate uncertainty, and speed up the repeated simulation loops that PBPK qualification demands [14,15,16,21].

This review has three aims. The first is to give a coherent account of the developments, organized not by network architecture but by the PBPK workflow each one enhances, namely virtual patient generation (Section 3), cross-scale extrapolation (Section 4), and parameter estimation with uncertainty quantification (Section 5). The second is to tie these methodological advances to a concrete pediatric dosing problem, using antiepileptic drugs (AEDs), namely lacosamide, lamotrigine, and oxcarbazepine, as a running application (Section 6). The third is to position the field within the broader artificial intelligence (AI)-in-pharmacometrics literature and state plainly what is genuinely new. Earlier reviews have surveyed AI for pharmacokinetic concentration prediction and therapeutic drug monitoring (TDM) [22,23], and a complementary review has catalogued machine learning opportunities in PBPK modeling [16]. Relative to those surveys, the present review differs in three ways. It covers the generative and scaling dimensions of PBPK together rather than prediction alone. It keeps the mechanistic model, not the prediction, as the object of learning. And it maintains throughout a distinction between methodological maturity, which the reviewed work demonstrates, and clinical validation, which it largely does not. Our contribution is a focused synthesis of virtual patients, learned allometry and ontogeny, and mechanistic parameter inference, with pediatric drug development as the organizing application. Throughout, the perspective is deliberately mechanistic.

Section 3 and Section 4, and 5 take up the three learning tasks in turn, Section 6 brings them to bear on pediatric AED dosing, Section 7 surveys the open-source ecosystem that makes the program reproducible, and Section 8 and Section 9 address open problems and the limitations of this review before Section 10 concludes.

## 2. The PBPK Framework and Its Bottlenecks

### 2.1. Mechanistic Structure of PBPK Models

A PBPK model rests on two layers. The physiological layer fixes, for a given virtual individual, body weight, organ and tissue volumes, regional blood flows, tissue composition (water, lipid, and protein fractions), and the expression and activity of drug-metabolizing enzymes and transporters in relevant organs. The drug layer fixes physicochemical properties (log P, pKa, molecular weight), plasma protein binding, blood-to-plasma partitioning, membrane permeability, tissue partition coefficients, and intrinsic clearances for each elimination pathway [3,4]. Mass-balance equations couple the two layers. For each organ compartment the rate of change in drug amount equals inflow minus outflow minus intrinsic elimination, with tissue partitioning handled through equilibrium partition coefficients or permeability-limited transport. Solving the resulting system of ordinary differential equations numerically yields concentration–time profiles in plasma and tissues. Both layers are coupled through the same mass-balance equations. The physiology is shared across species and ages; the drug parameters are not.

Two properties of this architecture matter for what follows. The physiological layer is largely drug-independent and shared across species and ages; the same equations describe a mouse, a human adult, and a neonate, differing only in physiological inputs, and that is the mechanistic basis of extrapolation. The drug layer, by contrast, is sparse. Many parameters (tissue partition coefficients for each organ, intestinal permeability, transporter kinetics) cannot be measured directly in humans and have to be predicted from in vitro data, animal studies, or physicochemical properties, each source bringing substantial uncertainty [3,16]. Extrapolation is possible because physiology is shared; uncertainty enters because the drug parameters are sparse and imprecisely known.

### 2.2. The Pediatric Extrapolation Workflow

Pediatric PBPK analysis follows a largely standardized workflow [9,10,24,25]. One begins with an adult PBPK model that has been developed and verified against adult clinical data. The model is then translated to pediatrics by substituting age-appropriate values in the physiological layer. The substitution draws on published age–weight–height growth charts, organ weight and blood flow ontogeny equations, and enzyme and transporter ontogeny functions for the pathways relevant to the drug [24,25]. Predicted pediatric exposure is then checked against whatever pediatric clinical data exist. Where agreement is acceptable, the model serves to simulate exposures across age bands and to pick doses that match the adult target exposure [9,10]. In essence, one builds the model in adults, swaps in pediatric physiology, and checks the predictions against available pediatric data. The procedure is simple in outline but demanding in practice.

At the quantitative heart of this workflow sits the ontogeny function. For each enzyme (CYP3A4, CYP2C9, UGT2B7) and transporter (OAT1, OAT3, P-glycoprotein), the pediatric-to-adult ratio of abundance or activity is modeled as a function of age. The usual form is a sigmoidal or piecewise exponential curve. It is fitted to proteomic or activity data that are scarce, heterogeneous, and often based on only a handful of donors per age band [6,24,26,27]. The pediatric ontogeny framework developed by Johnson and colleagues, which underpins the Simcyp population simulator, remains the most widely used implementation, and it established that physiologically based prediction of pediatric clearance outperforms simple allometry, most markedly in children under two years of age [28]. The developmental patterns themselves are well documented. Glomerular filtration rate matures quickly in the first weeks of life. CYP3A7, the dominant fetal CYP3A isoform, is replaced postnatally by CYP3A4. UGT2B7 and several sulfotransferase isoforms follow their own trajectories. These patterns materially alter the clearance of many pediatric drugs, yet current ontogeny functions capture them only partially [6,24,27,29].

### 2.3. Three Bottlenecks as Machine Learning Problems

Before proceeding, the three bottlenecks are restated in a form that makes their connection to deep learning explicit.

Bottleneck 1: virtual patient construction. Regulatory-grade pediatric simulation needs virtual populations that reproduce not just marginal distributions but also the multivariate correlations and whole-body coherence of physiological attributes [11,12,19]. Sampling marginals independently and enforcing constraints by rejection distorts exactly those joint properties. The details are taken up in Section 3.1. Generative modeling of the joint physiological distribution is the natural machine learning formulation (Section 3).

Bottleneck 2: scaling laws. Cross-species allometry assumes a power law Y = a·BW^b with an exponent near 0.75 for clearance, and the empirical failures of that assumption are not marginal. Simple allometry mispredicts human clearance of protein therapeutics and monoclonal antibodies by several-fold, because target-mediated disposition and FcRn recycling violate the power-law premise [30,31]. Fixed exponents also introduce systematic bias at the extremes of the pediatric weight range, notably in neonates and low-birth-weight infants, where weight-based allometry consistently overpredicts clearance of renally and metabolically cleared drugs [32]. The 0.75 exponent is itself contested. A reanalysis of 619 mammalian species favors 2/3 over 3/4 [33], and the scaling exponent shifts systematically with body size, taxonomic level, and physiological state [34]. Exponents turn out to be compound- and mechanism-dependent, and they fail outright for transporter-mediated clearance and for scaling to children [13,35,36]. Ontogeny functions, in turn, remain empirical fits to sparse data. Treating scaling as a continuous, data-driven learning problem, in species space and in age space, is a supervised regression on a structured biological manifold, and it is well suited to neural networks that embed allometric and developmental priors (Section 4).

Bottleneck 3: parameter identification and uncertainty. PBPK models are over-parameterized relative to the clinical data usually available, identifiability is limited, and uncertainty propagation through the full model is rarely quantified end to end [15,16,17]. Classical calibration is manual and slow, and that becomes prohibitive when sensitivity analyses and population simulations require thousands of model runs. Physics-informed neural networks and surrogate emulators recast parameter estimation as optimization with mechanistic residuals and make uncertainty quantification (UQ) a first-class output (Section 5).

Each bottleneck maps onto a distinct learning task, namely generation, regression on physiological manifolds, and inverse problems, and the deep learning toolbox fits these cleanly (Table 1). The next three sections take them up in turn.

## 3. Generative Construction of Virtual Patient Populations

### 3.1. The Virtual Patient as the Unit of PBPK Simulation

Every PBPK simulation starts from a population, that is, a set of virtual individuals. Each individual is described by a vector of physiological attributes, including age, sex, weight, height, organ volumes, blood flows, enzyme abundances, transporter activities, and renal function. In pediatric applications, the developmental stage these attributes imply also matters [11,12]. The quality of the virtual population sets the quality of the simulated exposure distribution and, in turn, the credibility of the dose recommendation. Two requirements dominate. Marginal fidelity means each attribute must be distributed as in the target population. Multivariate coherence means the attributes must be jointly plausible. Organ masses cannot sum to more than body weight. Cardiac output must bound the sum of organ flows. Enzyme abundances must correlate with age in developmentally consistent ways [11,19]. A population that gets the marginals right but violates the joint structure yields simulations whose variability is not physiologically meaningful.

Classical population construction is a sequential affair. Demographic sampling comes first, taking age and sex from census or trial data. Weight and height come next, from growth charts. Organ volumes and blood flows follow, from age-based equations. Enzyme abundances come last, from ontogeny functions. The user often specifies correlations by hand [12,25]. Constraints are enforced by rejection sampling. Workable as it is, the pipeline has well-known deficiencies. Correlation structure among physiological parameters is imposed piecewise and inconsistently. Rejection sampling is inefficient in high dimensions. The resulting populations tend to underestimate the true multivariate spread [11,12]. Underneath these practical problems sits a tacit independence assumption. Parameters are drawn from marginal distributions and then combined, with correlations patched in only piecewise. That construction systematically underestimates the tails of the joint distribution, the extreme but plausible combinations of organ sizes, flows, and enzyme abundances that marginal sampling cannot reproduce. These tails are not an academic curiosity. They are exactly where dose-related adverse events concentrate. An infant with simultaneously immature renal function, low metabolic capacity, and unusual body composition is a rare joint event. Marginal sampling will rarely generate it. Yet its simulated exposure is the most informative for safety. This is the argument that motivates the generative approach of Section 3, where marginal sampling is easy but modeling the joint distribution is the hard part.

### 3.2. Generative Models for Physiological Data

Generative modeling has contributed three families of methods to physiological and pharmacokinetic data analysis, namely variational autoencoders (VAEs), generative adversarial networks (GANs), and denoising diffusion probabilistic models (DDPMs). All three aim at the same objective, learning the joint distribution p(x) of a high-dimensional observation vector x from training data and then sampling new x’s. They part ways on how sample quality, training stability, mode coverage, and the ease of injecting domain constraints are traded off.

An autoencoder approach builds on a low-dimensional latent representation in which an encoder maps x to a latent z, and a decoder reconstructs x from z, with the evidence lower bound as the objective [40]. When auxiliary variables c (age, sex, and so on) are fed into both encoder and decoder, the model becomes a conditional VAE (cVAE) and gains controlled generation [11,41,42]. Training is stable and the latent space is smooth, which supports interpolation and targeted generation, although historically the samples have tended to be somewhat blurred. GANs, by contrast, pair a generator with a discriminator. They can yield sharp samples. They are notoriously hard to train, and mode collapse is a known failure [43]. Diffusion models take a third route, corrupting data with gradually increasing noise and then learning to reverse the corruption, sampling through iterative denoising [44]. This achieves sample quality comparable to the best current generative models and conditions naturally, at the cost of more expensive sampling.

Two additional considerations push physiological data toward particular architectures. Physiological parameters are bounded, correlated, and often roughly lognormal or skewed, so the generator must respect ranges and non-negativity. And the biological plausibility constraints of Section 3.1 need to be satisfied, ideally by construction or through a physics-informed loss rather than post hoc filtering. These considerations drive the recent turn toward physiology-informed generative models.

### 3.3. Physiology-Constrained Generative Models in PBPK

The clearest demonstration of physiology-constrained generation for pediatric PBPK is the physiology-informed conditional variational autoencoder (cVAE) of Irie and Mizuno ([11], preprint); [45]. Trained on real-world pediatric data augmented with mechanistically derived physiological information and conditioned on age and sex, the model generates body size, estimated glomerular filtration rate, organ weights, and blood flows across the full pediatric age range. Three design choices stand out. Physiological constraints were embedded directly in the training objective. The model was penalized for violating bounds and cross-attribute relations, such as total organ mass exceeding body weight or the sum of organ blood flows exceeding cardiac output, so that all generated profiles satisfied the constraints without rejection sampling. The latent space was then shown to encode smooth developmental trajectories, allowing targeted generation, for example a virtual infant with specified kidney maturity. The utility of the generated physiology was validated by feeding virtual patients into vancomycin PBPK simulations that reproduced reported age-dependent exposure trends ([11], preprint). Together these elements make the cVAE a practical blueprint, one that couples mechanism-informed constraints with a structured latent space and downstream PBPK validation.

At the level of next-generation PBPK frameworks, diffusion models have also been proposed. The physiologically informed multi-scale framework of Liu, Qiu and Wang couples a Foundation PBPK Transformer, which treats pharmacokinetic forecasting as sequence modeling and brings foundation-model scale to PK, with a Physiologically Constrained Diffusion Model (PCDM) that generates biologically compliant virtual patient populations ([18], preprint). The PCDM adds a physics-informed loss during denoising and, on synthetic benchmarks, reduces physiological violation rates from 2% to 0.5%. Evaluated on synthetic data only, the framework still articulates an architectural future in which a generative population layer produces outputs that are biologically admissible by construction and feeds downstream PBPK simulation at scale [18]. These results come from synthetic benchmarks only; the violation rate improvement has not yet been tested against clinical data.

Around these two anchors, the generative approach to virtual populations is being explored from adjacent directions. In silico trial communities have long relied on statistical copulas and Gaussian-process surrogates to build correlated virtual cohorts [39]; these are gradually giving way to deep generative alternatives that learn correlation structure from data instead of assuming a parametric form [16,46]. In rare-disease and pediatric settings, where training data are small, hybrid schemes that initialize a generative model from mechanistic equations and fine-tune it on sparse clinical data, mirroring the Sim2Real strategy in pharmacometrics ([47], preprint) between mechanism and data. A caveat applies across all of these demonstrations. None has yet been benchmarked head-to-head against classical population construction on the same clinical datasets, and the metrics reported so far (for instance, the reduction in physiological violation rates) reflect internal validation on generated or synthetic data. Do generative approaches translate into better downstream PBPK predictions than classical rejection-sampling pipelines? That remains an open empirical question. Only external validation against independent pediatric clinical exposure data can answer it. Table 2 compares the main generative approaches. The approaches are promising, but their clinical superiority over classical methods remains to be demonstrated.

### 3.4. Pediatric Virtual Populations: Developmental Structure as Inductive Bias

Pediatric physiology poses a distinctive generative challenge because the joint distribution is strongly structured by development. Age does not simply enter as a covariate; it organizes the entire distribution. Organ weights, enzyme abundances, and body composition follow age-dependent trajectories with sharp transitions at birth, weaning, and puberty, and the correlations themselves change with age (the age–weight relationship in infancy differs from that in adolescence) [1,6,11]. Generative models that condition on age capture this structure explicitly. The cVAE of Irie and Mizuno produced smooth latent developmental patterns by conditioning on age and sex [11], and the PCDM conditions the denoising process on physiological state [18]. This developmental conditioning is the generative analogue of the ontogeny function. Both encode the same biological knowledge; the difference is that the generative form learns joint correlations rather than marginal trajectories.

Data sparsity adds a further layer of difficulty. Pediatric physiological training data, from organ weights across ages to enzyme abundances per age band, are scarce and heterogeneously sourced [6,11]. Three mitigations recur in the literature. Real data can be augmented with mechanistically derived pseudo-observations, as in the cVAE study ([11], preprint). Known physiological relationships can be embedded as soft constraints so that the model cannot generate biologically impossible points even where data are sparse [11,18]. And transfer learning from adult physiology or from animal developmental data can stretch the data that do exist [16,48]. Together these make the generative pediatric population problem tractable today at proof-of-concept scale, and a central research target for the next few years. None of these mitigations removes the underlying data scarcity; they make the problem tractable at proof-of-concept scale.

## 4. Learning Cross-Species and Cross-Age Scaling Laws

### 4.1. Allometry and Ontogeny: Classical Foundations and Their Limits

Cross-species extrapolation of pharmacokinetic parameters rests on allometric scaling, the assumption that physiological rates and drug clearance scale with body weight as a power law, Y = a·BW^b [13,35,37]. Kleiber observed that basal metabolic rate scales with an exponent of roughly 0.75 [49]. West, Brown and Enquist later gave this observation mechanistic grounding through fractal network theory [50]. Together they made 0.75 the default clearance exponent in simple allometry. Volume of distribution scales near-linearly (b ≈ 1.0), and half-life near b ≈ 0.25 [13,35]. Correction schemes (maximum life-span potential, brain weight, the rule of exponents, liver blood flow corrections) were introduced to cope with compounds for which the simple exponent fails [35,51]. Pediatric scaling from adults uses the same power-law machinery within a species, with age-dependent weight. In many PopPK models the allometric exponent is either fixed at 0.75 or estimated from data [6,7]. A single assumed exponent necessarily ignores the many mechanisms that deviate from it.

Fixed-exponent allometry has well-documented limitations. The exponent is not a universal constant but compound- and mechanism-dependent. Transporter-mediated clearance and biliary excretion often deviate from metabolic scaling [35,36]. The 0.75 exponent is contested even as a description of metabolism [50,52]. Extrapolation from adults to children fails when a disposition pathway is absent or qualitatively different in early life, as with CYP3A7-to-CYP3A4 switching [6,24]. Ontogeny functions address the age dimension separately, but they are themselves piecewise empirical fits to sparse data, with known disagreements among datasets and fitting approaches [6,24,26]. What the field needs are scaling laws that are continuous, data-adaptive, mechanism-aware, and validated across both species and age.

### 4.2. Learned Scaling Laws: From Fixed Exponents to Data-Driven Maps

What deep learning offers, conceptually, is a shift from treating scaling as a single fitted exponent to learning a map from descriptors to disposition parameters. Before turning to specific architectures, it is worth distinguishing three extrapolation tasks that this literature often groups together, because evidence for one does not transfer automatically to the others. The first is cross-species extrapolation, inferring human disposition from animal or in vitro data. The second is cross-age extrapolation within a species, inferring pediatric disposition from adult data and developmental physiology, which is the task that matters for pediatric dosing and the one for which external validation is scarcest. The third is prediction of human pharmacokinetic parameters from molecular structure and nonclinical descriptors, a compound-oriented task that involves no age dimension at all. The subsections below treat each in turn, and for each we indicate whether the method has been evaluated on pediatric age extrapolation or only on an adjacent task.

Neural allometry via graph networks and neural ODEs. Its most explicit formulation is the Neural Allometry component of Liu, Qiu and Wang’s multi-scale framework, in which a graph neural network (GNN) coupled with a neural ordinary differential equation (neural ODE) learns continuous cross-species scaling laws ([18], preprint). The GNN captures the dependency structure among physiological variables, such as organ network connectivity. The neural ODE supplies continuous-time dynamics for parameter trajectories along the size and age axes [18,53]. Discrete structural relations are encoded by the graph, continuous scaling by the ODE. The pairing speaks directly to the nonlinear, rapidly changing nature of developmental physiology that fixed exponents cannot represent. Dynamic GNNs for PBPK, which model inter-organ interactions through recurrent message passing, have already performed strongly on multi-organ pharmacokinetic data (MAPE 15.7%, R2 = 0.9342) ([20], preprint), and the extension to scaling laws is natural, although that result concerns inter-organ prediction rather than age extrapolation. One technical caveat concerns the developmental axis. Neural ODEs are continuous-time models, whereas several pediatric transitions occur fast relative to the timescale over which model parameters are held fixed. Glomerular filtration rate rises steeply within the first weeks of life, and CYP3A7-to-CYP3A4 switching occurs over a narrow postnatal window [6,24]. These are rapid, highly nonlinear changes rather than true discontinuities, but a continuous model can still smooth over them unless they are represented explicitly. Whether a neural ODE captures such transitions depends on how they are encoded, either as time-dependent input functions, an age indicator or developmental-stage variable entering the network, or as stiffness in the governing equations that the solver treats as a steep but continuous change. Neither encoding happens automatically. Without an explicit developmental input, a continuous model will smooth over precisely the transition that dominates neonatal dosing. This is an open technical requirement for learned ontogeny, and we return to it in Section 8.3.

Multimodal and transfer approaches to human PK prediction. A complementary line of work replaces the allometric formula with machine learning models that predict human clearance and volume from molecular structure and nonclinical data, trained on historical compound databases [13,37,38]. These models match conventional allometry in accuracy (geometric mean fold errors of roughly 2.1–2.5 for clearance) and also learn mechanism-relevant structure, derived features that fixed exponents ignore [37,38]. For pediatric applications, transfer-learning variants that fine-tune an adult-trained PK model on pediatric data offer a data-efficient route to age-dependent prediction [16,48]. Reviews of human PK prediction now routinely frame the evolution as one from allometry to artificial intelligence, with the emerging consensus that AI complements rather than replaces mechanistic scaling [13,37].

Ontogeny as a learned function. Learning extends to the ontogeny function itself. Given proteomic or activity data across age, a neural network or Gaussian process can estimate the continuous developmental trajectory together with its uncertainty, replacing the piecewise fits of classical practice [6,24,26]. The learned ontogeny can then be embedded. The same network can also encode the constraint that ontogeny functions be smooth, monotonic where physiology requires it, and consistent across enzymes [26]. Learning ontogeny jointly with other physiological parameters, as in the cVAE latent space, further ensures that enzyme abundance correlates coherently with the rest of the virtual patient’s physiology [11]. Learning ontogeny as a continuous trajectory, rather than as a set of piecewise fits, is one of the clearest gains of this approach.

### 4.3. Mechanism-Aware Design: What Neural Scaling Preserves

What the from-allometry-to-AI literature teaches is that pure data-driven scaling fails outside the training distribution, and that is precisely the regime where extrapolation is most needed [13,37]. The physiologically constrained approaches reviewed here answer by preserving mechanism in the architecture, where the graph structure encodes which organs interact and the ODE dynamics encode mass balance, and physiological constraints are enforced in the loss [18,20]. We are nonetheless cautious about the word preserve. A GNN or neural ODE learns a dynamical map that is, at bottom, a black-box function approximation. Even when the architecture embeds mass balance, the learned parameters, such as a scaling exponent for clearance, are optimized for data fit, not for physiological identifiability. Nothing guarantees that the fitted map corresponds to a true biological mechanism, and a network can in principle exploit a non-physiological shortcut, a correlation present in the training data that does not generalize mechanistically, while still satisfying the constraint terms. Mechanism-inspired is therefore a more accurate label than mechanism-preserving. The architecture is inspired by physiology. What is preserved is the constraint structure, not necessarily the biological interpretation of the learned parameters. That distinction matters for validation (Section 8.1) and regulatory credibility (Section 8.2). It is the same design principle that has proved decisive in physics-informed PK modeling more broadly [14,54], now applied to the scaling dimension of PBPK. In short, the architecture is inspired by mechanism, the constraints are preserved by construction, and the interpretation of learned parameters requires care.

## 5. Parameter Identification and Uncertainty Quantification

### 5.1. The Inverse Problem in PBPK

PBPK models routinely contain dozens of parameters whose values are uncertain, including tissue partition coefficients, intestinal absorption parameters, and enzyme and transporter kinetics. Fitting these from sparse clinical concentration data is an inverse problem, made harder by structural non-identifiability (many parameter combinations produce the same plasma profile) and by the computational cost of repeated model evaluation [15,17]. Classical practice combines in vitro-to-in vivo extrapolation (IVIVE) priors with manual or semi-automated calibration [3,8]; uncertainty is usually reported for only a subset of parameters, and end-to-end propagation of parameter uncertainty to dose recommendations is rare [15,16,17]. With many parameters and few data, the inverse problem is the central difficulty in PBPK estimation.

### 5.2. Physics-Informed Neural Networks for PBPK

Physics-informed neural networks (PINNs) train a network to approximate the solution of a dynamical system while penalizing the residual of the governing equations in the loss [14]. In pharmacokinetics, PINNs have been shown to recover population-level parameter distributions from aggregated data (distributional PINNs, D-PINN) [21]. They match classical nonlinear least-squares estimators on compartmental models. They additionally recover unmeasured compartments and expose identifiability problems [15,55]. They also serve as the backbone of mechanism-constrained generative frameworks [18,54]. For PBPK specifically, PINN-based estimation has two advantages. The mechanistic ODE system (mass balance, dosing inputs) provides a strong prior that stabilizes estimation from sparse pediatric data [15,16,21]. The network’s ability to expose flat loss directions reveals which parameters the data can and cannot identify [15]. This diagnostic capability is not unique to PINNs. The same non-identifiability can be diagnosed with classical tools, and it is worth being precise about which property is at issue. Structural identifiability is a theoretical property of the model together with the experimental design, asking whether the parameters are uniquely determined by idealized, error-free observations, and it is established analytically rather than numerically. Practical identifiability asks whether the available data, with their noise and limited sampling, constrain the parameters well enough to estimate them usefully, and it is assessed through sensitivity analysis or through the Fisher information matrix (FIM), whose rank and conditioning reflect local parameter information content at a given operating point. Parameter uncertainty is a further step again, describing the spread of estimated or posterior parameter values once a statistical model has been assumed. FIM-based analysis therefore addresses practical rather than structural identifiability, and it is local rather than global. We therefore do not claim identifiability diagnosis as a PINN-specific capability. What is distinctive about PINNs is computational. By amortizing the forward solve, they make it practical to estimate full parameter posteriors, for example via distributional PINN outputs [21]. Parameter uncertainty can then be propagated through subsequent simulations within a Bayesian framework, at a fraction of the cost of Markov-chain Monte Carlo on the mechanistic model. Put plainly, PINNs do not reveal what FIM cannot. They make Bayesian uncertainty quantification computationally tractable, and that is their genuine contribution to PBPK.

### 5.3. Surrogate Emulation and Simulation Acceleration

Deep learning also works as a surrogate, a fast neural emulator trained to reproduce the input–output map of a full PBPK model and then used in place of the slow solver for Monte Carlo population simulation, sensitivity analysis, and Bayesian calibration [16,39,56]. Surrogates have been applied to quantitative systems pharmacology and to PBPK sensitivity workflows, cutting the cost of thousands of model runs to milliseconds per evaluation [39,56]. Coupling emulators with Bayesian updating, where posterior distributions over tissue partition coefficients and enzyme abundances are refined as preclinical and early clinical data accumulate, has been identified as one of the most defensible paths toward regulatory-facing hybrid PBPK–machine learning (ML) frameworks [8,16,37]. The speed gain is real, but an emulator remains an approximation of the mechanistic model and must be validated as such before its outputs are trusted.

### 5.4. Uncertainty Quantification as a Reporting Standard

Across generation, scaling, and estimation alike, the field is converging on a single principle. Point outputs are insufficient, because dose decisions are decisions under uncertainty [16,22,57]. The toolbox is substantial, but its components answer different questions and should not be treated as interchangeable. Distributional outputs and posterior PBPK parameter distributions (D-PINN) [21] quantify uncertainty in the mechanistic parameters themselves, the quantities a pharmacologist most wants to inspect. Bayesian neural networks and deep ensembles [57,58] quantify uncertainty in the learned mapping from inputs to outputs, which combines data noise with model misspecification and does not separate them. Conformal prediction [22,59] quantifies the calibration of predictive intervals in the output space and makes no claim about parameters at all, while posterior predictive checks [17] ask whether the mechanistic structure is consistent with the observed data. Calibrating a predictive interval is therefore not equivalent to quantifying uncertainty in a PBPK parameter, and a study that reports only the former has not shown that its dose recommendation is robust to physiological uncertainty. For generative virtual patients, the analogous requirement is reporting the coverage and calibration of generated physiological distributions against external data, as in the cVAE study’s distribution-matching evaluation ([11], preprint). We join recent reviews [16,22,37] in urging that UQ become a standard component of AI-enhanced PBPK publications, and we recommend that authors state explicitly which of these quantities they report. The regulatory dimension of this principle is taken up in Section 8.2. Point outputs cannot support dose decisions under uncertainty; the spread of the predicted exposure must be reported alongside the point estimate.

## 6. Application: Pediatric Precision Dosing of Antiepileptic Drugs

### 6.1. The Clinical Problem

Epilepsy affects roughly 0.5–1% of children, and antiepileptic drugs (AEDs) are among the most commonly prescribed drug classes in pediatric neurology [6,60,61]. Dosing the class is difficult. Therapeutic windows are narrow. Inter-individual pharmacokinetic variability is substantial, and the newer agents in particular carry limited pediatric labeling and sparse developmental pharmacokinetic data [6,60,62]. Three AEDs illustrate the range of challenges. Consider lacosamide, a third-generation AED that selectively enhances slow inactivation of voltage-gated sodium channels. It is approved in children from 4 years of age in the United States. The European Medicines Agency (EMA) indication was later extended to children from 2 years of age. Its oral bioavailability is near-complete, protein binding is low, and clearance is predominantly renal with a minor CYP2C19 component [5,7]. Lamotrigine is cleared mainly by glucuronidation (UGT1A4, UGT2B7). It responds strongly to enzyme induction by co-administered AEDs such as carbamazepine and phenytoin, and to inhibition by valproate. These interactions can shift clearance several-fold [63,64]. Oxcarbazepine acts through its active monohydroxy metabolite (MHD). It is cleared by glucuronide conjugation and renal excretion, and it induces its own metabolism [65,66]. All three are monitored in pediatric practice, yet dosing remains largely population-average, with individualization achieved mainly by trial and error [60,62,67].

From a PBPK standpoint the three drugs cover much of the developmental-physiology space. Renal maturation dominates lacosamide clearance in infancy [5,7]; UGT ontogeny and drug–drug interactions dominate lamotrigine [63,64]; and the active-metabolite kinetics of oxcarbazepine force one to model both parent and MHD across ages [65,66]. That diversity makes the AED class a natural test bed for the framework developed above.

The exposure target that any of these methods would serve deserves explicit statement, because it is an assumption rather than a measurement. Dose selection in this section is framed as matching pediatric exposure to adult exposure, and that framing rests on three assumptions. The first is that the exposure-response relationship established in adults transfers to children, that is, that the exposure associated with seizure control and with central nervous system toxicity in adults is the appropriate target in a child whose brain is still developing; this is the standard bridging assumption of pediatric drug development rather than a demonstrated equivalence [2,9]. The second is that the metric chosen for matching is the one that governs the response: total and unbound AUC for efficacy, Cmax for concentration-dependent toxicity, and, for oxcarbazepine, exposure to MHD rather than to the parent drug, since the antiepileptic effect resides in the metabolite [68,69]. The third is that the adult target remains valid under the co-medication that dominates variability in this population, given that enzyme inducers raise MHD clearance by about 25% [68] and lower lacosamide exposure by about 35% [7], while valproate inhibits lamotrigine glucuronidation and can roughly double exposure [63,64]. Where an antiepileptic drug has an established therapeutic reference range, the target can be checked against it independently of the adult-matching assumption, and simulations can report the fraction of the virtual population falling below, within, and above that range. Where no such range is established, the target remains a bridging assumption and should be reported as one, together with the metric, the dosing interval, and the unbound fraction on which it depends.

### 6.2. Existing PBPK and PopPK Evidence for Pediatric AEDs

Pediatric PBPK modeling of AEDs has come a long way. A Chinese population PBPK model of lacosamide was developed and verified against observed adult concentrations [5]. The verified adult model was then applied by simulation to pediatric and renally or hepatically impaired populations, producing age-band doses chosen to reproduce adult target exposure: 1.75 mg/kg intravenously in newborns up to 2 mg/kg in adolescents, and 20 mg orally in toddlers up to 95 mg in adolescents. Those pediatric doses are extrapolations from the adult-verified model: they were not evaluated against observed concentrations in each age band, and Table 3 marks that distinction explicitly [5]. That work exemplifies the classical adult-to-pediatric extrapolation workflow of Section 2.2. It also supplies a verified mechanistic scaffold onto which the deep learning layers of Section 3, Section 4 and Section 5 can be attached. Population pharmacokinetic models of lacosamide in children provide the clinical parameter estimates and variability structures that generative and scaling models can learn from and benchmark against. The underlying data also define the youngest age at which pediatric observations are available in the studies reviewed here: 402 concentration samples from 79 children aged 6 months to 17 years, with allometric weight scaling of clearance and a documented 35% decrease in average concentration under enzyme-inducing co-medication [7].

For lamotrigine and oxcarbazepine the pediatric PBPK picture is more complete than it was, but it still stops short of early infancy. A pediatric PBPK model of lamotrigine has been built in PK-Sim from a verified adult model and extrapolated to children aged 2 to 12 years and to patients with renal impairment, where it predicted the exposure thresholds associated with the doses recommended for initial and long-term treatment, and showed that severe renal impairment raises exposure about 1.5-fold [70]. Oxcarbazepine has been modelled with PBPK in children aged 2 years and older, for MHD and for the parent drug, and the model has been used to quantify how age and enzyme-inducing co-medication change MHD clearance; it also showed that children aged 2 to 4 years receiving maximum recommended doses are at risk of exceeding the reference exposure range [68]. The same structure has since been applied to children with and without obesity, and its evaluation against pooled trial data shows both what verification looks like in practice and how demanding it is: in the full dataset of 41 children only 53% of MHD concentrations and 23% of the parent-drug concentrations fell within the 90% prediction interval; MHD coverage reached about 86% only after 15 of the 41 children, whose concentrations were systematically under-predicted, were excluded in an exploratory analysis, a discrepancy the authors attributed to unrecorded disease state, genotype, or interacting medication [69]. Population pharmacokinetic studies in Chinese children supply complementary age-band models in which weight, valproate co-administration, and enzyme inducers are the dominant covariates [63,64,65,66].

The residual gaps are therefore specific rather than general. Both the lamotrigine and the oxcarbazepine PBPK models are verified only from two years of age upward, so the neonatal and infant bands remain extrapolation for two of the three drugs; lacosamide is the only one of the three for which pediatric doses have been simulated across the whole age range, from newborns to adolescents, but those pediatric predictions rest on extrapolation from an adult-verified model rather than on verification against neonatal observations [5]. UGT1A4 and UGT2B7 maturation is still poorly characterized in early infancy, and PBPK predictions in neonates are correspondingly uncertain [28,63,64]. For oxcarbazepine the formation of MHD from the parent drug and its subsequent glucuronidation have not been characterized in the first two years of life, which is also the period in which the fractional renal excretion of MHD changes fastest [68,69]. Table 3 lists representative modelling studies for the three drugs together with the clinical data used, and it distinguishes the age range over which each model has been evaluated against observed concentrations from the ranges reached only by simulation. What limits the whole class is the situation identified in Section 2.3: ontogeny functions are fits to sparse data, virtual populations are statistically crude, and extrapolation uncertainty is rarely quantified, and none of the pediatric PBPK models above reports a full uncertainty budget for the dose it recommends. It is important to be precise about what this evidence directly supports. Direct pediatric-AED evidence now comprises population PBPK models for lamotrigine and oxcarbazepine verified from two years of age upward, an adult-verified lacosamide model whose pediatric predictions span newborns to adolescents without neonatal verification, and the PopPK studies in Table 3, the youngest of which begin at 6 months. None of the mechanism-constrained methods of Section 3, Section 4 and Section 5 has yet been applied to pediatric AED data. Those methods are transferable to the AED setting in principle, but the transfer remains a research agenda, not an accomplished fact, a distinction we maintain throughout the rest of the review.

### 6.3. What Physiologically Constrained Deep Learning Offers to Pediatric AED Dosing

Mapping the three methodological threads onto the AED problem gives a concrete research agenda.

#### 6.3.1. Generative Pediatric AED Populations

The physiology-informed cVAE blueprint ([11], preprint) can be instantiated with AED-specific physiological conditioning. A virtual pediatric epilepsy cohort has to reflect not only age–weight–organ structure but also disease-associated covariates, including epilepsy comorbidity profiles, co-medication patterns, and renal function distributions. Generated populations would then feed PBPK simulations of lacosamide, lamotrigine, and oxcarbazepine to yield dose–exposure distributions across age bands, with physiological coherence guaranteed by construction [11,18]. For oxcarbazepine the generative layer would extend naturally to parent–metabolite co-distribution, a dimension classical population construction handles poorly.

#### 6.3.2. Learned Ontogeny and Scaling for AED Disposition

Because AED clearance is pathway-specific, AEDs are natural candidates for learned scaling. Lamotrigine (UGT), oxcarbazepine (UGT plus renal), and lacosamide (renal plus CYP2C19) exercise different ontogeny functions, and a neural ontogeny model trained jointly across pathways could exploit shared developmental structure while keeping pathway-specific trajectories intact [18,24,26]. Cross-species neural allometry, trained on AED preclinical data (rodent, dog, monkey) and fine-tuned on pediatric clinical data, offers a route to earlier human and pediatric predictions for AEDs still in development [18,37,38].

#### 6.3.3. Mechanistic Parameter Inference for AED PBPK

PINN-based estimation [15,21] and surrogate emulation [39,56] can update AED PBPK parameters from sparse pediatric TDM data, the very data assets built by hospital TDM services [67]. They also expose identifiability limits. For instance, they show how strongly pediatric plasma data constrain the ontogeny of UGT1A4 for lamotrigine. Bayesian emulation would then propagate parameter uncertainty into dose recommendations. This converts point-dose guidance into risk-bounded recommendations [16,57].

#### 6.3.4. Which Parameters Pediatric Data Support and Which Must Be Estimated

The three drugs differ in how much of their disposition is informed by pediatric data, and that difference determines what a dosing application would actually rest on. For lacosamide the data-supported elements are the adult-verified scaffold, the renal fraction of clearance, and the maturation of glomerular filtration, which is among the best characterized developmental processes relevant to drug elimination [5,7,24]; the parameters that would still have to be estimated are the distribution of renal function in infants and the contribution of CYP2C19 to clearance in early life, because plasma concentrations in the first months of life are sparse. For lamotrigine the data-supported elements are smaller: clearance is governed by UGT1A4 and UGT2B7 glucuronidation, and the models reviewed above establish that body weight and co-medication, specifically valproate and enzyme inducers, dominate the variability [63,64,70]. What pediatric data do not settle is the maturation trajectory of UGT1A4 in the first year of life, so the ontogeny function enters the dose calculation as a prior to be estimated jointly with the data or else propagated into the result, and lamotrigine is precisely the case in which a learned rather than a fitted ontogeny function changes the predicted exposure [28]. For oxcarbazepine the gap is structural as well as developmental: the parent drug is reduced by cytosolic aldo-keto reductases to MHD, and MHD is eliminated partly by UGT1A9 and UGT2B7 glucuronidation and partly by renal excretion of unchanged MHD, with a fractional renal excretion estimated at about 0.3 in the published model [68,69]. Formation and elimination are both age-dependent and were estimated jointly, so separating them would require paired parent and metabolite concentrations with urinary recovery, a dataset pediatric studies rarely produce. The consequence for dose selection is the same in all three cases. A parameter supported by pediatric data may be used with its estimated uncertainty attached. A parameter that is not supported must be treated as a prior whose uncertainty is propagated rather than ignored. Where a pathway is only jointly identified, the recommendation should be reported as conditional on the assumed split between formation and elimination, and the sensitivity of the recommendation to that assumption should be stated. Section 6.4 sets out the sensitivity analysis this requires.

### 6.4. Worked Example: From an Adult Lacosamide PBPK Model to a Pediatric Virtual Population

A worked example makes the preceding discussion concrete and shows where the difficulties actually lie. Lacosamide is a suitable vehicle for three reasons. A verified mechanistic scaffold already exists, since the Chinese population PBPK model of lacosamide was developed against adult data and then translated to pediatric and renally or hepatically impaired populations [5]. Its clearance is predominantly renal, and glomerular filtration rate has one of the best-characterized developmental trajectories of any process relevant to drug elimination [7,24]. Pediatric PopPK estimates are available for benchmarking, based on 402 samples from 79 children aged 6 months to 17 years [7].

The workflow has five steps, each corresponding to one of the methodological components reviewed above. The first is to fix the mechanistic scaffold, that is, the adult-verified structure, the drug-specific parameters such as partition coefficients, fraction unbound, and the renal fraction of clearance, and the physiological layer expressed so that age and population are inputs rather than estimated quantities [3,4]. The second is to construct the pediatric virtual population. Instead of sampling marginals, a constraint-parameterized generative model conditioned on age and sex produces virtual children whose organ masses, blood flows, and renal function satisfy hard physiological bounds jointly, in the manner of the physiology-informed cVAE [11]. The third is to replace the piecewise ontogeny fits for renal maturation with a learned continuous function carrying monotonicity and smoothness constraints, so that the maturation trajectory is differentiable with respect to age and its uncertainty can be propagated [6,24,26]. The fourth is to update the parameters that the pediatric data actually inform, using PINN-based estimation or a surrogate emulator on whatever sparse concentration data exist, returning a posterior distribution rather than a point estimate [15,21,39]. The fifth is to run Monte Carlo simulation across the virtual population and report, for each age band, the exposure distribution and the dose that attains the adult target exposure together with its credible interval.

Three differences from the classical workflow of Section 2.2 define the methodological contribution. The virtual population is generated jointly rather than assembled from marginals, so the tails of the physiological distribution are represented. The developmental input is a learned continuous function rather than a table of piecewise fits. Parameter uncertainty is propagated into the dose recommendation rather than being reported for a subset of parameters. The opportunities that follow are correspondingly specific. Because the generative model is conditioned on age, the range of ages over which its output is supported by training data can be stated explicitly, which converts an implicit assumption into a documented limit. Because uncertainty is propagated, the parameters that dominate the variance of the recommended dose can be identified, and that ranking indicates where additional sampling would be most valuable, whether in a neonatal physiology study or in routine therapeutic drug monitoring.

The sensitivity of the predicted exposure to an input error is not a property of the platform but a drug-specific quantity, and the lacosamide example makes the arithmetic explicit. Let f be the fraction of total clearance that depends on renal function. With nonrenal clearance, dose, and bioavailability unchanged, a proportional bias b in renal clearance changes total clearance by approximately f times b, and therefore changes exposure by approximately f times b divided by (1—f times b) in the opposite direction. For lacosamide about 40% of a dose is recovered unchanged in urine, so f is approximately 0.4, and a 20% under-estimate of renal function produces an 8% reduction in total clearance and an 8.7% increase in exposure. Only renal clearance of the parent drug enters this calculation. Urinary recovery of the O-desmethyl metabolite is a downstream fate of drug that has already been cleared metabolically, so it does not contribute to renal clearance of lacosamide itself and cannot be added to f. The same reasoning applies to metabolic inputs: a bias in an ontogeny function matters in proportion to the fraction of parent clearance that the enzyme handles, and that fraction must be established for the parent rather than inferred from the fate of its metabolites.

Input-error propagation therefore has to be analysed rather than asserted, and we propose it as a three-stage exercise. The first stage is local: compute the elasticity of the target exposure to each physiological and drug-specific parameter. For renal function that elasticity is exactly the fraction f above, which means the same bias in the same input can be benign for one drug and clinically relevant for another. The second stage is global: decompose the variance of the predicted exposure over the joint distribution of the inputs, using variance-based indices or a screening design, because the contributing errors are not independent and the propagation is nonlinear once virtual populations, ontogeny functions, and parameter posteriors are combined. The third stage carries the result into the decision: simulate across a Monte Carlo sample of virtual patients and parameter posteriors, and report the dose that attains the target exposure as an interval together with the parameters that dominate the variance of that dose, so that a recommendation whose interval spans a clinically relevant exposure range is reported as such rather than as a number. In neonates and infants the binding limits are the maturation data themselves. Glomerular filtration in the first weeks of life is described by ontogeny functions fitted to small series, and published functions disagree most in exactly that window [6,24,26,28], while UGT1A4 maturation rests on a limited number of in vitro abundance measurements, which is why lamotrigine predictions in newborns carry wider uncertainty than their point estimates suggest [28,63,64]. Where the propagated interval spans the therapeutic range, the appropriate conclusion is that the simulation cannot support a dosing decision in that age band, and we would rather state that limit than report a dose the data cannot defend.

The limitations are equally concrete, and they are the reason we do not present this workflow as an accomplished system. Neonates between birth and one month are the age band in which maturation is fastest and dosing is most error-prone, and it is also the band in which pediatric physiological reference data are thinnest. A generative model cannot manufacture the missing information, so virtual neonates should be regarded as extrapolated rather than supported. The renal maturation function dominates early-life predictions, and its own uncertainty is largest in exactly that window, which means the propagated uncertainty will be widest where clinical decisions matter most. Without an external pediatric cohort, the simulated exposure distributions cannot be independently confirmed, and agreement with the single-centre PopPK estimates used here would be a consistency check rather than a validation. All of this also inherits the quality of the reference physiology, because if the underlying organ-mass and blood-flow distributions are biased for a given population, the generated virtual patients will reproduce that bias faithfully. The example therefore shows how the components compose and, at the same time, why the validation and data questions of Section 8 are prerequisites rather than afterthoughts.

### 6.5. Toward an Integrated Virtual Pediatric AED Platform

**Figure 1:**
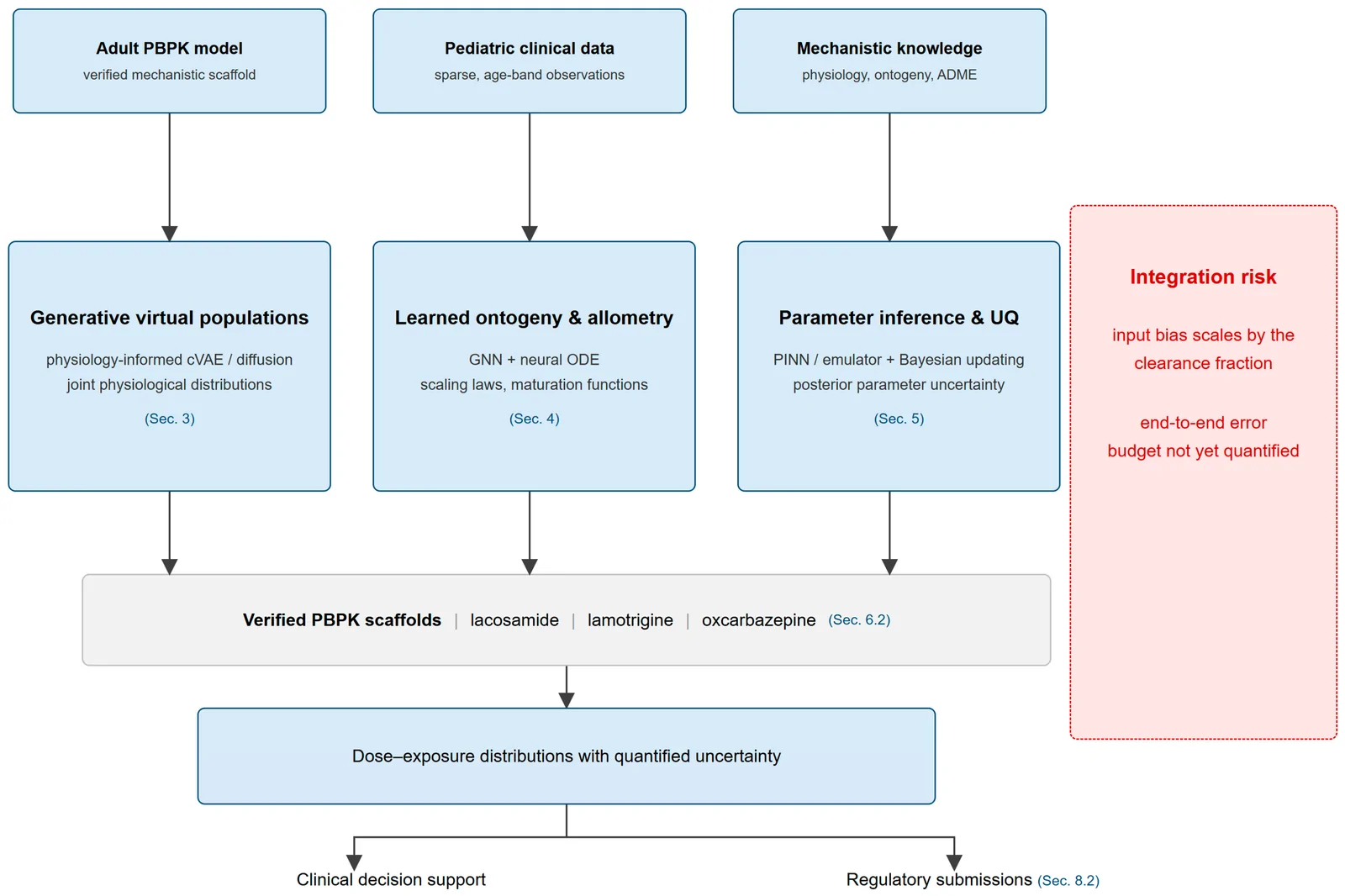
Proposed architecture of a physiologically constrained deep learning platform for pediatric AED precision dosing. Inputs, on the left, are the verified adult PBPK model, pediatric clinical data, and mechanistic knowledge of physiology, ontogeny, and ADME. The three methodological components of Section 3, Section 4 and Section 5 feed the PBPK scaffolds reviewed in Section 6.2, whose output is dose-exposure distributions with quantified uncertainty. The right-hand panel lists the integration risks discussed in Section 6.5.

Much of what follows is a proposal rather than an accomplished system. The PBPK scaffolds reviewed in Section 6.2 have been evaluated against observed concentrations in adults and, for lamotrigine and oxcarbazepine, from two years of age upward; the pediatric predictions for lacosamide extend from newborns to adolescents but have not been verified in the first months of life. The generative, scaling, and inference components (Section 3, Section 4 and Section 5) remain at proof-of-concept stage. These three threads converge on an integrated platform. It combines a generative virtual pediatric epilepsy population (Section 3), linked to learned ontogeny and allometry (Section 4) and to PINN/emulator parameter inference (Section 5). It runs the PBPK scaffolds for lacosamide, lamotrigine, and oxcarbazepine, keeping the age ranges evaluated against observed data distinct from those reached by simulation (Section 6.2 and Table 3). The output is dose-exposure distributions with quantified uncertainty, for clinical decision support and regulatory submissions. Thanks to the open-source ecosystem of Section 7, such a platform is technically constructible today. The principal remaining barriers are validation standards (Section 8.1) and the regulatory pathway (Section 8.2). Figure 1 illustrates the proposed architecture. Integration itself carries risks that the individual proof-of-concept components do not yet resolve. Errors propagate across modules. Under linear pharmacokinetics an input bias does not amplify: a small bias in the generated virtual population, say a 5% under-estimate of renal function in infancy, propagates into exposure with an elasticity set by the fraction of clearance that renal function governs, as Section 6.4 sets out. For lacosamide, where that fraction is about 0.4, such a bias moves exposure by about 2%, so amplification should not be assumed. Amplification would require a specific mechanism, such as an input acting on a nonlinear disposition pathway, a bias large enough to move a patient across a therapeutic or dose boundary, or several independent biases aligned in the same direction, and none of these has been demonstrated for a pediatric antiepileptic drug. What remains genuinely uncharacterized is the end-to-end error budget when generative error, scaling error, and parameter uncertainty are concatenated, since that propagation has not been quantified for any integrated platform. Quantifying that propagation, and benchmarking the integrated platform against external pediatric clinical data, is a precondition for any regulatory-facing use. We flag it as the most important open engineering problem.

## 7. Open-Source Ecosystem and Reproducibility

### 7.1. Mechanistic Platforms

The Open Systems Pharmacology (OSP) Suite (version 12), comprising PK-Sim and MoBi, is the flagship open-source PBPK platform. It is GPL-licensed, ships with built-in human and preclinical physiology databases and pediatric extrapolation workflows, offers an R/Excel interface, and includes a Qualification Framework with published qualification reports for pediatric extrapolation (CYP2C8/3A4/GFR ontogeny), DDI, and special populations [3,4]. OSP is also the natural host for the deep learning integrations reviewed here. Generative population outputs can be imported as virtual populations, and learned ontogeny functions can be supplied as physiological inputs. nlmixr2 and mrgsolve provide complementary open-source PopPK modeling in R, useful for benchmarking learned scaling against classical estimates [71].

### 7.2. Data Resources

Training data for the generative and scaling models come from several open sources. MIMIC-IV, an anonymized EHR with drug administrations and laboratory values, has become a de facto benchmark for AI-in-TDM research [72]. Pediatric physiological reference datasets, including organ weights, body composition, and enzyme abundance compilations published by OSP and others, supply the developmental information [4,11,24]. And Therapeutics Data Commons (PyTDC) offers an AI-ready suite spanning ADME, DDI, and toxicity tasks [73]. For AED-specific work, published pediatric PopPK datasets (lacosamide [7]; lamotrigine [63,64]) and hospital TDM archives [67] are essential complements. A recurring theme in the reproducibility literature is that pediatric physiological data remain fragmented across publications; harmonized, versioned, open compilations are a high-value community target [11,16,24]. Without such harmonized compilations, the field will keep re-assembling the same scattered datasets study by study.

### 7.3. Method Codebases

Methodology, too, is increasingly open. The physiologically informed multi-scale framework (Foundation PBPK Transformer, PCDM, Neural Allometry) comes with reproducible implementations ([18], preprint). Dynamic GNN PBPK code is public [20]. D-PINN and chemotherapy-PINN implementations are available [15,21]. The vancomycin_dose_predictions pipeline ships MIMIC-IV extraction plus LSTM and gradient-boosting training code [74]. Irie and Mizuno’s physiology-informed cVAE remains a proof-of-concept with data and code available on request [11]. Following OSP community practice, we recommend that future method papers release versioned, archived code, for example on Zenodo, alongside manuscripts. This keeps the field’s reproducibility guarantees intact [3,4]. Table 4 summarizes the key open resources. The community’s willingness to share code is a precondition for the reproducibility guarantees discussed above.

## 8. Challenges, Open Problems, and Future Directions

### 8.1. Validation of Generative Virtual Populations

Most pressing among the open problems is validation, namely how to certify that a generated virtual population is fit for regulatory-grade simulation. Following the cVAE study and related work [11,16], we identify three testable criteria. Distributional fidelity requires that generated marginals, and above all correlations, match independent external reference data. Constraint satisfaction requires that hard physiological constraints hold for every generated individual, not merely on average. Downstream validity requires that PBPK simulations run on generated populations reproduce observed exposure trends in external clinical data, as in the downstream exposure validation of the cVAE-generated population. Standardized benchmarks, meaning shared pediatric physiological datasets, fixed constraint lists, and agreed metrics, are needed so that different generative architectures can be compared [11,16]. This mirrors the broader trajectory of AI for drug discovery, where benchmark discipline has been decisive [73]. Certification of a generated population depends on all three criteria.

The criteria are only useful if they are operational, so we specify how each would be assessed and on what data. Verification is against independent pediatric concentration data, and the first decision is which data may serve as verification. Data that informed parameter estimation cannot also serve to test the model, so the cohorts, age bands, and sampling windows that inform it must be declared in advance and separated from those used to evaluate it, whether by an external cohort, by a temporal split, or, when only published datasets are available, by a study-level split. The verification report then has two levels. At the level of the concentration-time profile, prediction-corrected visual predictive checks should show the observed 5th, 50th, and 95th percentiles falling within the simulated intervals, with the proportion of observations inside the 90% prediction interval reported per age band rather than pooled, because pooling conceals the youngest band, where performance is weakest. The pediatric oxcarbazepine evaluation is a useful precedent because it reports both an overall result and an exploratory subset analysis. In the full dataset of 41 children, 53% of MHD concentrations and 23% of parent-drug concentrations fell within the 90% prediction interval; MHD coverage reached about 86% only after 15 of the 41 children, whose concentrations were systematically under-predicted, were excluded from the exploratory analysis. Reporting both figures, rather than the higher one alone, is what makes the extent of the remaining problem visible [69]. At the level of exposure, the metrics compared should be the ones the decision actually uses, namely steady-state AUC, Cmax, and trough concentration, reported as fold-error distributions and as absolute average fold error against a pre-specified limit rather than as a single average [68,69].

An acceptance criterion is a statement about the clinical consequences of being wrong, and it should be written on the exposure scale. Under linear pharmacokinetics AUC is inversely proportional to clearance, so for an antiepileptic drug with a narrow separation between the exposure that controls seizures and the exposure that produces central nervous system toxicity, a 20% under-estimate of clearance produces a 25% over-estimate of AUC and a 20% over-estimate produces a 16.7% under-estimate. The same proportional reasoning applies to average steady-state concentration, but not directly to Cmax or to trough concentrations, which depend on the absorption and elimination rate constants as well as on clearance. The clinically relevant question is whether an error of that size moves a patient across a decision boundary, from an adequate dose to an inadequate one or from a therapeutic exposure to a toxic one. Acceptance limits should therefore be tied to the width of the therapeutic range and to the granularity of the dose increments that can actually be prescribed, and they should be tighter in the age bands where dosing is most error-prone than in adolescents. The choice of metric matters for the same reason: a model that reproduces mean exposure within 10% but misplaces the upper tail of the distribution can still generate a dose that is unsafe for the patients that tail represents.

Improvement over the conventional workflow, finally, has to be demonstrated rather than assumed, and the appropriate test is a paired comparison. The conventional pediatric PBPK workflow, with fixed ontogeny functions and a manually assembled virtual population, and the learning-enhanced workflow should be run on the same data against the same acceptance criteria, with the difference in fold error and in interval coverage reported by age band. The claim this review makes is that learning-enhanced workflows should help most in the youngest bands, where conventional ontogeny extrapolation is weakest; if a head-to-head comparison does not show that pattern, the added complexity is not justified. No such comparison has yet been published for a pediatric antiepileptic drug, and we regard it as the single most informative experiment the field could run next.

Three patterns are apparent from Table 5 and are worth stating explicitly. First, direct pediatric evidence is concentrated in the classical PBPK and PopPK rows, which are peer-reviewed and use real clinical data but contain no learning component. Second, the methods with the strongest methodological claims, namely generative virtual populations and learned scaling laws, are supported mainly by rows classified as (ii) or (iii), several of which are preprints or lack an external comparator. Third, external validation is rare at every level: only three rows report validation against data outside the development setting, and only one of these addresses a pediatric population. This is the empirical basis for the distinction between methodological maturity and clinical validation that we maintain throughout the review.

### 8.2. Regulatory Qualification of AI-Enhanced PBPK

Model-informed drug development is already accepted by the U.S. Food and Drug Administration (FDA) and EMA for PBPK and PopPK analyses [8,9,10], but that acceptance should not be read as extending automatically to the learning components discussed in this review. Classical PBPK earned its regulatory standing through mechanism transparency, traceability of physiological inputs, and prospective validation against observed data, properties that a regulator can audit [3,4,8]. A generative or neural component embedded in the same workflow raises a distinct question, namely whether its specific output can be trusted for a defined context of use, and answering that question requires a credibility assessment of the component rather than an endorsement of the platform that hosts it [4,8,16]. Two developments look poised to help. The OSP Qualification Framework provides a community-reviewed template for model qualification [3,4], and recent regulatory initiatives favoring human-relevant prediction methods, such as the FDA’s 2025 roadmap to reduce animal testing in preclinical safety, raise the demand for credible in silico alternatives while making this distinction more, not less, important [13,37]. Generative virtual populations and learned scaling laws, if validated per Section 8.1, are natural candidates for qualification within this framework. A recurrent regulatory question concerns how the uncertainty emitted by a PINN or generative model differs from the parameter variability and model residual that classical PBPK already reports. These three quantities are not interchangeable. Classical parameter variability captures inter-individual differences within a known structure. Model residual captures the unexplained gap between model and data. ML-native uncertainty, such as posterior spread or conformal intervals, is calibrated only to the training distribution and potentially overconfident outside it. In practice, the least-trusted element of an AI-enhanced submission is likely not the neural architecture itself but the training data, whether the virtual population, ontogeny functions, and learned scaling laws were derived from data that cover the intended pediatric context of use. We therefore argue that data representativeness, not black-box-ness, is the binding constraint on regulatory acceptance, and that transparent reporting of training-data coverage (per Section 8.3) will matter more than architectural interpretability.

### 8.3. Data Scarcity, Identifiability, and Multi-Scale Integration

Before considering methodological remedies, it is worth stating plainly that the binding constraint on pediatric model-informed precision dosing is not algorithmic but empirical. Every generative model, learned scaling law, and parameter estimator discussed in this review is limited by the quality, quantity, and reliability of the data used to train and validate it. Pediatric physiology is characterized by small samples at every level. Reference values for organ weights, blood flows, and enzyme abundances derive largely from post-mortem and surgical series with only a handful of donors per age band, which is why reported ontogeny functions disagree even for well-studied enzymes [6,24,26]. Clinical pharmacokinetic data are sparse by design, since ethical and practical constraints limit both the number of children enrolled and the number of samples per child, and the resulting datasets often cannot support estimation of the full parameter set of a mechanistic model. Routine therapeutic drug monitoring generates large volumes of concentration data, but these records are heterogeneous, inconsistently structured, and fragmented across institutions, so they are rarely reusable as model-development assets [22,67]. The consequence deserves emphasis: a more expressive architecture cannot substitute for absent measurements. Better algorithms reorganize the information available, they do not create it, and in the pediatric setting the information available is thin precisely where it is needed most, namely in neonates and infants, where maturation is fastest. Mitigation therefore has to be partly infrastructural rather than purely computational. Federated learning across hospital TDM services [22], shared pediatric physiology reference databases, prospective opportunistic sampling built into routine care, and systematic reuse of existing trial data all address the input side of the problem, and we regard them as prerequisites for the methodological agenda rather than as optional complements to it.

Pediatric physiological data remain the binding constraint [11,24]. Beyond the mitigation strategies surveyed in Section 3.4, federated learning across hospital TDM services [22] is a further active direction. Identifiability, exposed rather than hidden by PINN-style methods [15], must be reported as a matter of course. Integration across scales, from generative physiology to learned scaling to verified drug models, requires software architecture as much as methodology; the OSP-plus-learning stack described in Section 7 is a plausible target. A related question concerns the extrapolation boundary. If an age band, say 2–6 years, contributes no training data, where does the generative model’s validity end? Our position is that validity must be stated explicitly as a function of the conditioning variables. Models trained on sparse pediatric data should report the support of the training distribution. Predictions outside that support should be flagged as extrapolation rather than interpolation. Mechanistic constraints (mass balance, monotonic ontogeny) narrow the extrapolation region without eliminating it, and validation against external age-band data remains the only honest certificate [11,16]. Given how sparse pediatric data remain, extrapolation should always be labeled as such.

### 8.4. Foundation Models and Digital Twins of Pharmacokinetics

The multi-scale framework of Liu, Qiu and Wang articulates the frontier. It combines foundation-scale transformer models of pharmacokinetics, generative populations, and learned scaling laws, unified under physiological constraints ([18], preprint). Such a foundation model would require a very large pretraining corpus. Once realized, it would be pretrained on multi-source corpora, including EHRs, clinical trials, mechanistic simulations, and physiological references. It would then be fine-tuned per drug and per population, with mechanism constraints retained to prevent plausible-but-wrong dynamics. A further step extends this to the individual patient. The patient becomes a continuously updated computational representation of a child’s physiology and drug disposition that drives real-time dose adjustment [75,76]. Pediatric AED dosing is a natural proving ground for such a model. It combines sparse sampling, high stakes, and a well-defined developmental axis [6,67]. Realizing this vision will require managing training bias in adult-over-represented corpora, catastrophic forgetting on small fine-tuning cohorts, computational footprint, and the ethical dimensions of automated dose changes. These risks must be designed for from the start [22,76]. Two further issues deserve attention. The first is architecture choice for spatio-temporal coupling. PK data are time series, and physiological parameters carry an explicit organ-level structure. A pure transformer may not capture this coupling naturally. A graph transformer or a spatio-temporal graph network, which respects the organ network as an inductive bias, may do better. We regard graph-based sequence architectures as a promising alternative for the Foundation PBPK Transformer and encourage empirical comparison on the multi-scale tasks of Section 4. The second issue is the ethics of automated dose adjustment in pediatrics. Legal responsibility for a dose recommendation ultimately rests with the prescribing clinician, not with the model that generated it. An AI-advised dose change raises questions of liability and clinical authority that the field has not yet answered. Who audits the model? Who documents the rationale? How does the clinician retain final decision rights when the recommendation is opaque? These governance questions are as consequential as the technical ones. They argue for keeping the human in the loop as a structural, not cosmetic, feature of any future platform. The ambition is considerable, the optimal architecture remains an open question, and the ethical framework is not yet settled.

## 9. Limitations of This Review

This review was compiled through a targeted rather than a systematic literature search, and we state its main features here so that the process is auditable. We searched PubMed, Web of Science, Scopus, arXiv, bioRxiv, and medRxiv for records published between January 2019 and August 2026, running two primary query streams. The first combined terms for machine learning architectures with terms for pharmacokinetics, namely (deep learning OR machine learning OR neural network OR graph neural network OR neural ordinary differential equation OR variational autoencoder OR diffusion model) AND (pharmacokinetic OR physiologically based OR drug concentration OR precision dosing). The second targeted mechanism-constrained work, namely (physics-informed OR scientific machine learning OR physiologically constrained OR physiologically informed OR mechanism-constrained OR neural ODE) AND (pharmacokinetic OR pharmacometric OR physiologically based OR precision dosing). Reference lists of included records were screened to capture foundational anchors published before 2019, among them the origins of allometric scaling, physiological ontogeny functions, and the PINN framework. Records were screened on title and abstract and then on full text; inclusion required that a study either developed or evaluated a computational method addressing virtual population construction, cross-species or cross-age scaling, or mechanistic parameter estimation, and reported at least one quantitative performance or validation result. Studies were excluded if they were purely descriptive, reported no quantitative evaluation, or addressed concentration prediction without any mechanistic component. Four principles guided purposive selection: representativeness of both methodological streams, methodological significance over incremental applications, diversity of evidence types, and extractability of quantitative detail. Applying these criteria to the retrieved literature yielded thirteen primary methodological studies, each graded individually in Table 5, alongside the pediatric PBPK and PopPK evidence summarized in Table 3, and the review cites 76 references in total. Coverage of clinical pediatric AED trials and of regulatory case reports is therefore illustrative rather than exhaustive.

Beyond the scope of the search, four limitations qualify the conclusions of this review. The first concerns the evidence base itself. Several key contributions reviewed here, most notably the physiology-informed cVAE [11] and the multi-scale framework [18], were available only as preprints at the time of writing. Their conclusions have been cited with appropriate caution and should be re-verified against the peer-reviewed versions once published. The status of these preprints was re-checked on 16 September 2026; each is labelled as a preprint in the reference list, and any that has since appeared in a peer-reviewed journal will be cited in its published form at proof stage. Quantitative comparisons across generative, scaling, and estimation methods are further limited by the heterogeneity of datasets, comparators, and metrics reported in the primary literature, which is why we describe performance numerically only where a study stated its own comparator. The second concerns uncertainty in model inputs. Physiological parameters such as organ blood flows, enzyme abundances, and transporter activities enter PBPK models with measurement error that is rarely reported, and generated virtual populations inherit and may amplify that error; a generative model trained on imperfect reference physiology will reproduce its biases faithfully rather than correct them. The third concerns the physiological assumptions that constrain the models. Mass balance, monotonic ontogeny, and plausible organ-size relationships are necessary but not sufficient for mechanistic correctness, and a model can satisfy every constraint while still misrepresenting the developmental biology that matters clinically. The fourth concerns external validation. Very few of the reviewed methods have been evaluated on data from an independent centre or age band, so the field-level claims made here should be read as assessments of methodological maturity rather than as benchmarks. The proposed pediatric AED platform (Section 6.5) is, for the same reason, a research agenda grounded in published scaffolds, not an implemented or clinically validated system.

## 10. Conclusions

PBPK modeling carried pediatric dose selection from empirical scaling to mechanism-based extrapolation. Deep learning now offers a second transition. It moves from hand-built physiological populations, fixed scaling exponents, and manual calibration toward generative virtual patients, learned allometry and ontogeny, and machine-inferred parameters with quantified uncertainty. Individually, the three threads sit at proof-of-concept maturity. Together they compose a coherent research program anchored in the mechanistic model rather than in concentration prediction. As an application context, pediatric precision dosing of lacosamide, lamotrigine, and oxcarbazepine is both a clinical need and a methodological stress test. It exercises renal, UGT, and active-metabolite disposition across the full developmental axis. Because of the open-source ecosystem (OSP, nlmixr2, MIMIC-IV, PyTDC, public method codebases), the program is reproducible at near-zero cost. One distinction deserves to be explicit, because the strengths of this approach are real but not equally well supported. Physical consistency and interpretability follow from the architecture by construction rather than from empirical demonstration. Improved parameter identifiability is documented in studies with an explicit comparator, chiefly against nonlinear least-squares estimation and against Fisher information analysis [15,21]. Evidence for improved extrapolation is weaker and more uneven. It is reasonably firm for cross-regimen and cross-species settings, and weakest for direct pediatric age extrapolation, where only a small number of studies have reported external age-band validation and several key results rest on preprints or on synthetic-data evaluation [11,18,20,47]. No reviewed method has yet demonstrated clinical superiority in real pediatric dosing decisions, because head-to-head comparisons on identical cohorts are essentially absent. We therefore state the claim in the form the evidence supports: physiologically constrained learning improves the structural and methodological properties of PBPK models, and has shown promising but, for pediatric extrapolation, still preliminary accuracy gains. We do not claim that the reviewed methods have been shown to improve extrapolation to untested pediatric ages, and none of them has yet been applied to pediatric antiepileptic drug data at all. A mature, mechanism-constrained dosing framework for pediatric AEDs therefore does not yet exist; we present one as a design target and a proving ground. The open problems are validation standards, regulatory qualification, and the discipline to report uncertainty. What will determine the field’s next phase is less algorithmic novelty than validation discipline and open science. Those same qualities carried classical PBPK to regulatory acceptance and now extend to its learning-enhanced successor. Demonstrations at proof-of-concept scale do not yet amount to validated products. The immediate priorities are validation against independent pediatric data and sustained open-science practice.

## Figures and Tables

**Table 1:** Mapping of PBPK bottlenecks to deep learning tasks.

PBPK Bottleneck	Deep Learning Task	Representative Approaches	Section
Virtual patient construction	Generative modeling of joint physiological distributions	Physiology-informed cVAE [11]; PCDM diffusion [18]	3
Cross-species/cross-age scaling	Regression on physiological manifolds (scaling laws)	Neural Allometry (GNN + Neural ODE) ([18], preprint); dynamic GNN [20]; ML human PK prediction [37,38]	4
Parameter identification & UQ	Inverse problems with mechanistic residuals	PINN/D-PINN [14,15,21]; surrogate emulation + Bayesian updating [8,39]	5

**Table 2:** Generative approaches for virtual patient populations compared.

Approach	Correlations Learned	Constraint Handling	Data Requirement	Pediatric Developmental Conditioning	Representative Work	Evidence Status and Key Limitation
Statistical sampling + rejection	Manual/piecewise	Rejection (inefficient)	Reference datasets	Separate ontogeny functions	Classical PBPK practice [12,25]	Established practice; limitation: distorts the joint physiological structure, and rejection sampling is inefficient in high dimensions
Copula/Gaussian-process surrogates	Parametric	Post hoc filtering	Moderate	Limited	In silico trial communities [12,39]	Used in in silico trial communities; limitation: the correlation structure is parametric and not physiologically constrained
cVAE	Learned (latent)	Physics-informed loss (hard)	Moderate; augmented with mechanistic data	Age/sex conditioning; smooth latent trajectories	Irie & Mizuno 2026 [11]	Demonstrated for pediatric virtual patients [11]; limitation: available as a preprint, one dataset, no external cohort validation
GAN	Learned	Adversarial + post hoc	Large	Possible via conditioning	Generative drug discovery [43,46]; synthetic clinical patients [45]	Demonstrated for synthetic clinical data but not yet for virtual-patient physiology [43,45,46]; limitation: training instability and mode collapse
Diffusion (PCDM)	Learned	Physics-informed denoising loss	Large; synthetic pretraining viable	State conditioning	Liu, Qiu & Wang 2026 [18]	Proposed for PBPK within a multi-scale framework ([18], preprint); limitation: no published virtual-patient evaluation, high sampling cost

**Table 3:** Representative pharmacokinetic modeling studies for pediatric AEDs (lacosamide, lamotrigine, oxcarbazepine). The third column states the clinical data and age range evaluated against observed concentrations, as distinct from the age ranges reached only by simulation.

Drug	Model Type	Cohort/Data Evaluated Against Observed Concentrations	Key Findings	Reference
Lacosamide	PBPK (Chinese population)	Adults (Chinese), evaluated against observed concentrations; pediatric dose recommendations obtained by simulation, from newborns to adolescents, without evaluation against observed pediatric concentrations	Age-band IV/oral doses to match adult exposure; renal dosing adjustments	Zhu et al. 2024 [5]
Lacosamide	PopPK	402 samples from 79 children aged 6 months to 17 years	Allometric weight scaling; 35% ↓ average concentration with enzyme inducers	Winkler et al. 2019 [7]
Lamotrigine	PopPK (age-group models)	473 Chinese children, 625 trough samples (age band not specified in the source)	Age-band-specific models; weight, valproate, inducers as covariates	Zhang et al. 2017 [63]
Lamotrigine	PopPK–pharmacogenetic	179 Chinese children (age band not specified in the source)	UGT2B7 genotype, valproate, oxcarbazepine effects on CL/F	Chen et al. 2018 [64]
Oxcarbazepine	PopPK (MHD)	Pediatric cohorts (age band not specified in the source)	Body weight allometry; maturation functions	[65,66]
Lamotrigine	PBPK (PK-Sim)	Adults, extrapolated to children aged 2 to 12 years and to patients with renal impairment (observed concentrations reported within the 5th–95th prediction interval)	Exposure thresholds for the recommended initial and maintenance doses; AUC increases about 1.5-fold in severe renal impairment	Li et al. 2025 [70]
Oxcarbazepine	PBPK (PK-Sim; MHD and parent)	Children aged 2 years and older, with and without enzyme-inducing co-medication	Age-dependent MHD clearance; enzyme inducers raise MHD clearance by about 25%; children aged 2–4 y may exceed the reference exposure range at maximum doses	Sinha et al. 2022 [68]
Oxcarbazepine	PBPK (MHD and parent)	41 children aged 2 to 20 years; 148 paired OXC/MHD samples; with and without obesity (full dataset: 53% of MHD and 23% of parent concentrations within the 90% prediction interval)	At least 80% of MHD concentrations within the 90% prediction interval for about two thirds of the children; AFE 0.85–0.98	Maglalang et al. 2026 [69]

**Table 4:** Key open-source platforms, datasets, and codebases for physiologically constrained PBPK learning.

Resource	Type	Role in This Review
Open Systems Pharmacology (PK-Sim/MoBi, version 12) [3,4]	PBPK platform	Mechanistic scaffold; host for learned populations/ontogeny; qualification framework
nlmixr2/mrgsolve [3,71]	PopPK (R)	Benchmarking learned scaling; complementary modeling
MIMIC-IV [72]	EHR dataset	Training/validation data for AI-PK components
Therapeutics Data Commons (PyTDC) [73]	AI-ready datasets	ADME/DDI benchmarks; transfer learning corpora
vancomycin_dose_predictions [74]	Method codebase	MIMIC-IV extraction + LSTM/ML pipelines (template)
cVAE (Irie & Mizuno) [11]	Method codebase	Pediatric virtual patient generation blueprint
Multi-scale PBPK framework [18]	Method codebase	Foundation PBPK Transformer + PCDM + Neural Allometry

**Table 5:** Evidence map for the methodological studies discussed in this review, classified by the strength of what each study has actually demonstrated.

Study (Ref)	Evidence Class	Task	Data Type and Size	Model Class	Comparator	Validation Type	Reported Performance	Pediatric Data	Peer-Review Status
Irie and Mizuno [11]	(i)	Pediatric virtual population generation	Real-world pediatric physiology; vancomycin exposure data	Conditional VAE with constraint-penalized loss	Reported age-dependent exposure trends	External (downstream PBPK)	Distribution matching; age trends reproduced	Yes	Preprint
Liu, Qiu and Wang [18]	(iii)	Multi-scale framework: foundation transformer, PCDM, Neural Allometry	Multi-source corpora proposed	Foundation transformer with diffusion and GNN-ODE components	Not reported	Internal	Not reported	Framework target	Preprint
Dynamic GNN PBPK [20]	(ii)	Multi-organ PK prediction	Multi-organ PK data	Dynamic graph neural network	Not reported	Internal split	MAPE 15.7%, R2 = 0.9342	No	Preprint
PINN parameter recovery [14,15]	(ii)	Parameter recovery and identifiability	Compartmental PK datasets	PINN with mechanistic residual	Nonlinear least squares; Fisher information analysis	Internal	Matches least squares; exposes flat loss directions	No	Journal
Tsiros et al. [21]	(ii)	Population parameter estimation	Aggregated population data	Distributional PINN	Classical estimation	Internal	Population parameter distributions	No	Journal
Baaz et al. [42]	(ii)	Population model estimation	PopPK datasets	VAE with neural ODE	Nonlinear mixed effects	Internal	Not quantified in this review	No	Journal
Hammann and Schoning [45]	(ii)	Synthetic patient generation	Clinical PK datasets	Wasserstein GAN	Classical sampling	Internal	Realism of generated patients	No	Journal
Sim2Real-PK [47]	(ii)	Dosing model transfer	Preclinical data; YODA individual patient data	Pretraining with fine-tuning	Standard dosing regimens	External (YODA IPD)	F50 and F20 coverage	No (adults)	Preprint
Mallillin et al. [37]	(ii)	Human PK parameter prediction	Historical compound databases	Machine learning versus allometry	Conventional allometry	Internal	GMFE about 2.1 to 2.5 for clearance	No	Journal
Chinese lacosamide PBPK [5]	(i)	Pediatric dose selection	Adult and pediatric clinical data	Classical PBPK	Adult target exposure	External across age bands	Age-band-specific doses	Yes	Journal
Winkler et al. [7]	(i)	Pediatric PopPK	402 samples from 79 children	Nonlinear mixed effects	None	Internal	Parameter estimates; 35 percent lower concentration with inducers	Yes	Journal
Lamotrigine PopPK in Chinese children [63,64]	(i)	Pediatric PopPK covariate analysis	Chinese pediatric cohort	Nonlinear mixed effects	None	Internal	Weight, valproate, and inducer covariates	Yes	Journal
MHD PopPK [65,66]	(i)	Active-metabolite PK	Pediatric cohort	PopPK with allometry and maturation functions	None	Internal	Parent and metabolite kinetics	Yes	Journal

Evidence class: (i) method demonstrated on a PBPK or PBPK-adjacent model with a stated comparator; (ii) adjacent PK or pharmacometric method, transferable to PBPK in principle but not yet demonstrated on it; (iii) proposed or future application. Fields marked “not reported” were not stated in the primary study, and we have not inferred or filled in missing values. GMFE, geometric mean fold error; MAPE, mean absolute percentage error.

## Data Availability

This review is based on published literature; the datasets and software analyzed are cited in the reference list. The open-source platforms and codebases discussed in Section 7 are available from the sources indicated therein.
